# Involvement of tRNA thiolation in uORF-mediated translational regulation during Xylogenesis in *Arabidopsis thaliana*

**DOI:** 10.1093/pcp/pcag055

**Published:** 2026-04-28

**Authors:** Yuichi Nishii, Daichi Araki, Mitsuru Saraumi, Taku Takahashi

**Affiliations:** Graduate School of Environmental, Life, Natural Science and Technology, Okayama University, 3-1-1 Tsushimanaka, Kitaku, Okayama 700-8530, Japan; Graduate School of Environmental, Life, Natural Science and Technology, Okayama University, 3-1-1 Tsushimanaka, Kitaku, Okayama 700-8530, Japan; Graduate School of Environmental, Life, Natural Science and Technology, Okayama University, 3-1-1 Tsushimanaka, Kitaku, Okayama 700-8530, Japan; Graduate School of Environmental, Life, Natural Science and Technology, Okayama University, 3-1-1 Tsushimanaka, Kitaku, Okayama 700-8530, Japan

**Keywords:** *Arabidopsis*, mRNA translation, thermospermine, tRNA thiolation, uORF

## Abstract

Post-transcriptional modification of tRNAs is an important mechanism for regulating translation efficiency and cellular homeostasis, yet its contribution to upstream open reading frame (uORF)-mediated translational control remains largely unexplored. In this study, we investigated the role of tRNA thiolation in thermospermine-dependent regulation of xylem development in *Arabidopsis thaliana*. Using a suppressor screen of the thermospermine-deficient mutant *acaulis5 (acl5)*, which exhibits dwarfism and excessive xylem differentiation, we identified *suppressor-of-acl502* (*sac502*) as a recessive loss-of-function allele of *CTU2*, a gene encoding a key enzyme in the biosynthesis of the wobble uridine modification 5-methoxycarbonylmethyl-2-thiouridine. Mutations in other components of the same modification pathway, including *ROL5* and *TRM9*, similarly suppressed the *acl5* phenotype. Translational analyses using 5′ leader-GUS reporter constructs revealed that the *ctu2* mutation did not enhance translation of the mRNA containing a thermospermine-responsive uORF of *SAC51*, but instead significantly reduced translation of that of *SACL3*, a member of the *SAC51* family, and that of *LONESOME HIGHWAY (LHW)*, which contains another conserved uORF in the 5′ leader region. Polysome profiling further demonstrated decreased association of *SACL3* and *LHW* mRNAs with actively translating ribosomes in *ctu2*. Genetic interaction analyses supported the conclusion that the suppression of excessive xylem formation in *acl5* by *ctu2* is attributable to reduced *LHW* activity. In addition, *ctu2* mutants displayed increased sensitivity to exogenous thermospermine, resembling the response of *lhw* mutants. Together, our results reveal that tRNA thiolation contributes to uORF-mediated translational regulation of key developmental regulators and identify tRNA modification as an important regulatory layer controlling vascular development.

## Graphical Abstract

**Figure:**
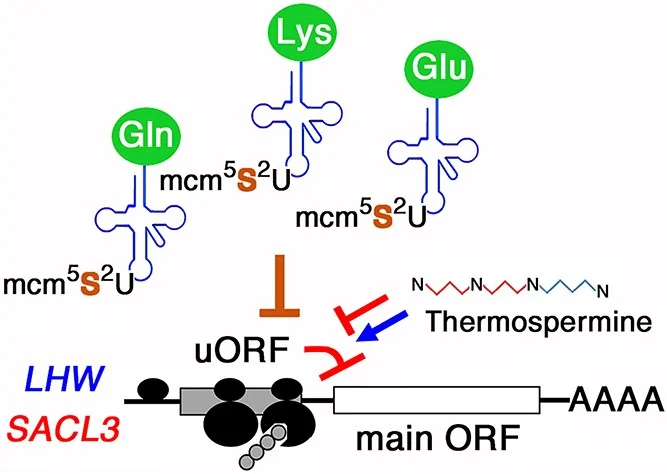

## Introduction

Post-transcriptional modifications of tRNAs are now recognized as critical regulators of translation efficiency, accuracy, and cellular homeostasis (Suzuki 2021, Phizicky and Hopper 2023, Zhang et al. 2025). More than one hundred distinct tRNA modifications have been identified across organisms, affecting base pairing, structural stability, and interactions with ribosomes and aminoacyl-tRNA synthetases (Cappannini et al. 2024). In plants, emerging evidence suggests that tRNA modifications play key roles in adaptive responses to fluctuating environmental conditions such as temperature changes, nutrient limitation, and oxidative stress, which require rapid and precise adjustments of protein synthesis modulating codon-specific translation, stress tolerance, and metabolic reprogramming (Wang et al. 2017, Zheng et al. 2024, Cai et al. 2025). In *Arabidopsis thaliana*, at least 21 types of tRNA modifications have been identified, and the studies have been rapidly expanding (Wang et al. 2017, Bhat et al. 2025).

These modifications occur at nearly all positions within the tRNA molecule but are particularly enriched at functionally important regions such as the anticodon loop and the tRNA core. Modifications at the anticodon loop, especially at the wobble position (U34) and the adjacent position (U37), influence codon-anticodon interactions, maintenance of the reading frame, and translational fidelity (Weixlbaumer et al. 2007). In contrast, modifications within the tRNA body contribute to proper folding, stability, and interactions with aminoacyl-tRNA synthetases (Björk et al. 1987). Among the modifications surrounding the anticodon loop, 5-methoxycarbonylmethyl-2-thiouridine (mcm^5^s^2^U) represents one of the most evolutionarily conserved chemical alterations (Björk et al. 2007). The mcm^5^s^2^U modification occurs at U34 of specific tRNAs, including tRNA-Lys (UUU), tRNA-Glu (UUC), and tRNA-Gln (UUG).

The precursor modification 5-carbamoylmethyluridine is first introduced at the C5 position of wobble uridine by the Elongator complex, a multi-subunit assembly composed of six core components, ELP1–ELP6 (Huang et al. 2005). This intermediate is subsequently methylated by the methyltransferase TRM9 to form mcm^5^U (Leihne et al. 2011, Iñigo et al. 2016). The thiolation pathway depends on the coordinated actions of several enzymes. In *Arabidopsis*, these include the cysteine desulfurase NFS1, the ubiquitin-related proteins URM11 and URM12, and sulfur transfer enzymes such as CNX5, ROL5, and CTU2 (Nakai et al. 2012, Philipp et al. 2014). Together, these proteins form a sulfur relay system that transfers sulfur derived from cysteine, replacing the oxygen atom at the C2 position of uridine to generate the fully modified nucleoside mcm^5^s^2^U (Nakai et al. 2019). In yeast, nematodes, and the rice blast fungus, *Magnaporthe oryzae*, loss of mcm^5^s^2^U modification causes ribosome pausing at AAA, CAA, and GAA codons, leading to disruption of proteome homeostasis (Rezgui et al. 2013, Nedialkova and Leidel 2015, Zhang et al. 2025). In *Arabidopsis*, defects in tRNA thiolation do not cause lethality but are associated with reduced lateral root density, abnormal leaf development, and impaired immune responses (John et al. 2014, Philipp et al. 2014, Nakai et al. 2019, Zheng et al. 2024). The *rol5* mutation has also been reported to act as a suppressor of the *leucine-rich repeat extensin 1* mutant, which displays abnormal root hair morphology, likely through changes in cell wall composition (Leiber et al. 2010). In rice, tRNA thiolation has been shown to be important for thermotolerance (Xu et al. 2020). Collectively, these findings suggest that tRNA thiolation serves as a key regulatory factor linking specific signaling pathways and metabolic states in plants.

We have studied the mode of action of a polyamine, thermospermine, in repressing xylem development in *Arabidopsis* (Takano et al. 2012). Thermospermine is produced specifically in the vasculature through auxin-induced expression of *ACAULIS5* (*ACL5*), which encodes thermospermine synthase. The *acl5* mutant shows dwarf and xylem-excess phenotypes. Studies on suppressor mutants of *acl5* (*sac*) have revealed that thermospermine enhances mRNA translation of the *SAC51* family genes, each of which contains a conserved upstream open reading frame (uORF) (Imai et al. 2006, Cai et al. 2016). Here we show that the loss of function of *CTU2* is responsible for one such suppressor mutant, *sac502*. The SAC51 family, a class of basic helix-loop-helix (bHLH) proteins, interferes with dimerization of the members of the LONESOME HIGHWAY (LHW) family with those of the TARGET OF MONOPTEROS5 family, which play a critical role as a bHLH transcription factor in auxin-induced xylem proliferation and also regulate the expression of *ACL5* (Katayama et al. 2015, Vera-Sirera et al. 2015). Namely, the transcription factor LHW and its family members, which function as an accelerator of xylem proliferation, are counteracted by the *SAC51* family acting as a brake, while thermospermine, under the control of the LHW family, participates as a negative feedback factor that appropriately reinforces this effect. The *LHW* mRNA also contains another uORF conserved within the family (Hayden and Jorgensen 2007). Our results suggest a critical involvement of thiolated tRNAs in the translational control of these conserved uORF-containing mRNAs.

## Results

### *sac502* is a recessive allele of *CTU2*

**Figure 1:**
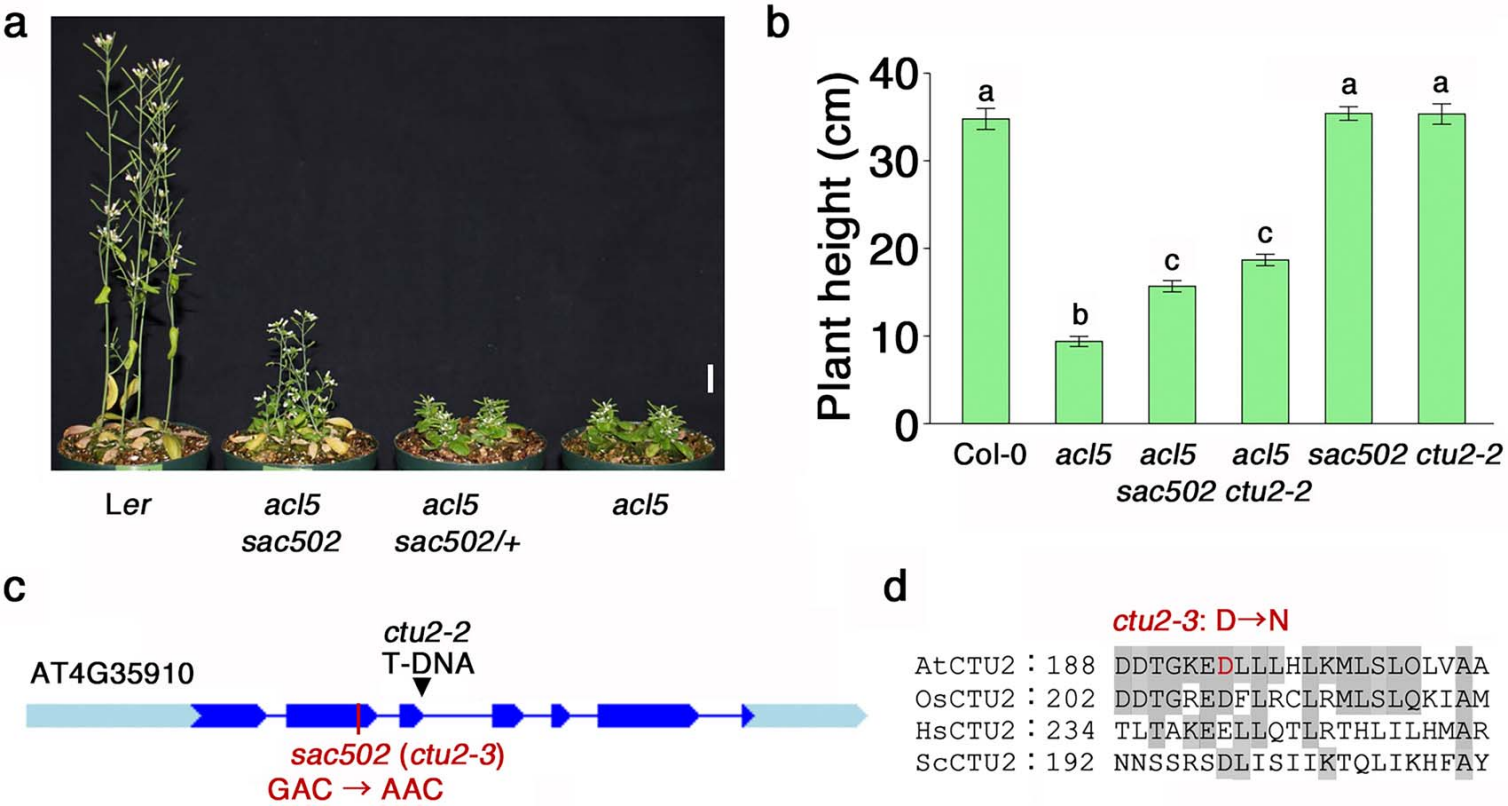
The dwarf phenotype of *acl5* is suppressed by mutations in *CTU2.* (a) Growth phenotype of 40-day-old wild-type (L*er*), *acl5* homozygous for *sac502, acl5* heterozygous for *sac502* (*sac502/+*), and *acl5* single mutant plants. Bar = 1 cm. (b) Plant height comparison between wild-type (Col-0) and mutant plants in the Col-0 background. Plants were grown in rockwool for 40 days. Mean ± SE, *n* = 5. Different letters indicate significant differences at *P* < 0.05 by ANOVA. (c) Structure of the *CTU2* gene. Protein coding regions and untranslated regions are shown in dark and pale pentagonal bars, respectively. (d) Alignment of amino acid sequences around the *sac502* (*ctu2-3*) mutation site with the corresponding sequences of the homologous proteins of different organisms. Amino acids identical to those in Arabidopsis are shaded. At, *Arabidopsis thaliana* (NP_567993); Os, *Oryza sativa* (XP_015618182); Hs, *Homo sapiens* (NP_001012777); Sc, *Saccharomyces cerevisiae* (NP_014280).

To identify factors involved in thermospermine-regulated xylem development, we have isolated suppressor mutants that restore the dwarf phenotype of a thermospermine-deficient mutant, *acl5*, through ethyl methanesulfonate (EMS) mutagenesis of *acl5* seeds (Imai et al. 2006, Kakehi et al. 2015). *sac502* was identified as a suppressor mutant that partially but significantly recovers the plant height of *acl5* (Fig. 1a and b). When original *sac502 acl5* plants in the L*er* accession were crossed with *acl5*, the F1 plants showed no suppression of the *acl5* phenotype, indicating that *sac502* is a recessive allele (Fig. 1a and b). Fine mapping combined with whole-genome sequencing revealed that *sac502* carries a single nucleotide substitution in *CTU2* (Fig. 1c), resulting in an amino acid change from aspartic acid, which is conserved across different plant species, to asparagine (Fig. 1d). Because a T-DNA insertion allele of *CTU2* has been previously reported as *ctu2-2* (Philipp et al. 2014), we examined whether *ctu2-2* also has the same effect as *sac502* on the *acl5* phenotype and confirmed a partial suppression of the dwarf phenotype of *acl5* by *ctu2-2* (Fig. 1b), suggesting that *sac502* represents a loss-of-function mutation of *CTU2*. We thus designated *sac502* as the *ctu2-3* allele (Fig. 1c). Hereafter, we mainly used *ctu2-2* for further experiments.

**Figure 2:**
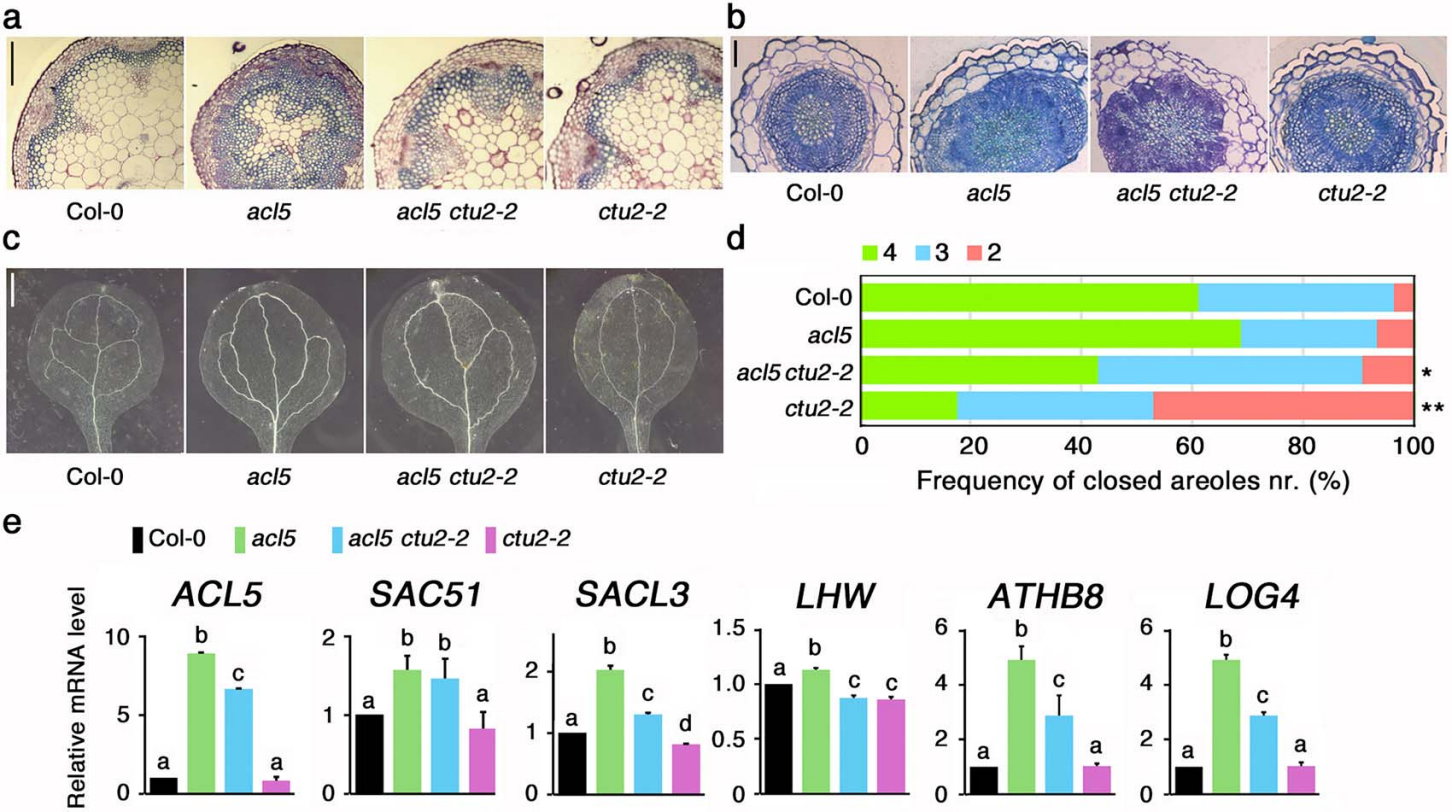
Suppression of *acl5* by *ctu2* at cellular and molecular levels. (a) Cross sections of the inflorescence stem of Col-0, *acl5, acl5 ctu2-2*, and *ctu2-2* plants. Stems were collected from the region ~2 cm above the roots of 20-day-old plants grown on rockwool. Bar = 200 *μ*m. (b) Cross sections of the hypocotyl of 20-day-old plants grown on rockwool. Bar = 200 *μ*m. (c) Comparison of cotyledon vein patterns of 7-day-old seedlings*.* Bar = 0.5 mm. (d) Quantification of closed areoles of cotyledon veins of 7-day-old seedlings*. n* = 30. Asterisks indicate significant difference from Col-0 (^*^P < 0.05, ^**^P < 0.01, Pearson’s chi-squared test). (e) Transcript levels of *ACL5, SAC51, SACL3, LHW, ATHB8*, and *LOG4* in 7-day-old wild-type (Col-0), *acl5, acl5 ctu2-2*, and *ctu2-2* seedlings. The levels were *normalized* to the *ACT8* transcript level and set to 1 in Col-0 (−). Means ± SE, *n* = 6. Different letters indicate significant differences at *P* < 0.05 by ANOVA.

In terms of xylem development, we confirmed by inflorescence stem sections that the excess xylem phenotype of *acl5* is apparently recovered by *ctu2-2* (Fig. 2a). Xylem differentiation in the *ctu2-2* single mutant was comparable to that in the wild type. A similar suppressive effect of *ctu2-2* on xylem development in *acl5* was also evident in the hypocotyls of mature plants (Fig. 2b). Examination of the cotyledon vasculature in seedlings showed that the thick veins of *acl5* were not markedly suppressed by *ctu2-2*. However, the areoles of the cotyledon veins were reduced in the *ctu2-2* single mutant and restored in the *acl5 ctu2-2* double mutant (Fig. 2c and d). The effect of *ctu2-2* on gene expression levels was examined in young seedlings by quantitative Real-Time PCR (qRT-PCR). Our results revealed that, among the genes examined, transcript levels of *ACL5, SACL3, ATHB8*, and *LOG4*, which are up-regulated in *acl5*, were reversed by *ctu2-2* but the level of *SAC51* was not (Fig. 2e). We detected a slight but significant reduction in the level of *LHW* transcripts by *ctu2-2.*

### Knockout of *ROL5* or *TRM9* also partially suppresses *acl5*

**Figure 3:**
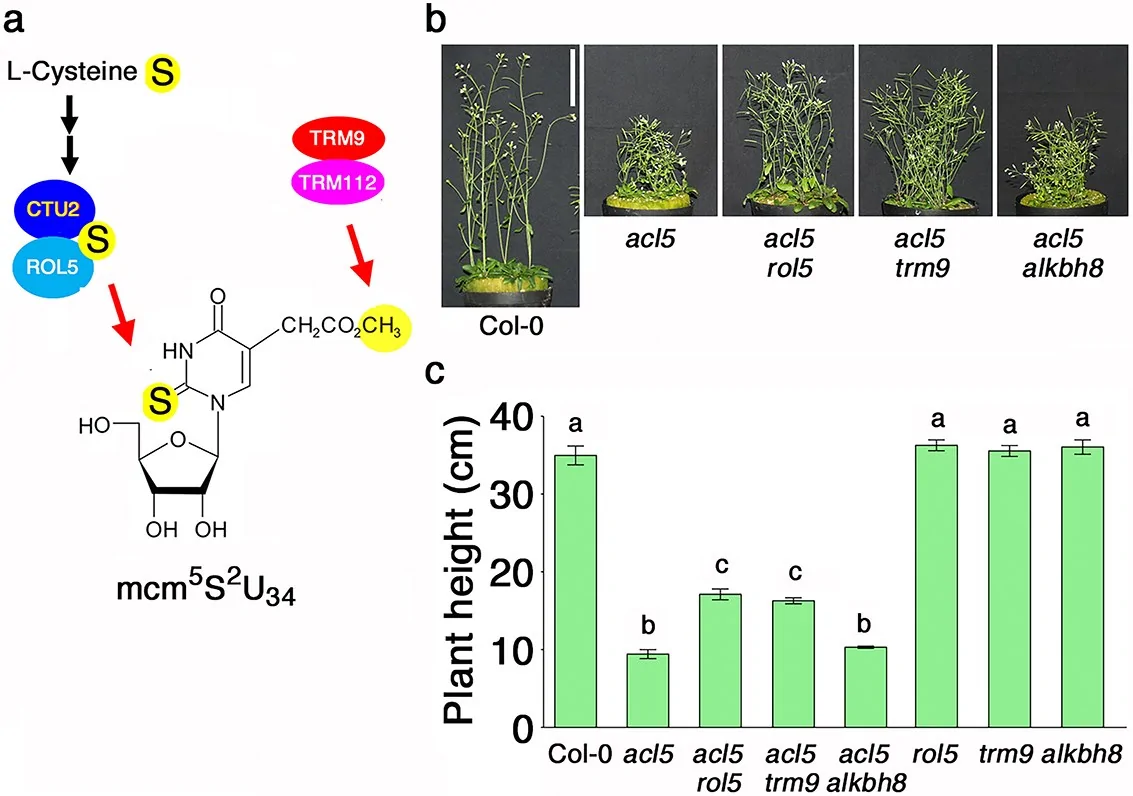
The dwarf phenotype of *acl5* is suppressed by defects in the mcm^5^s^2^U tRNA modification. (a) A schematic flow of the mcm^5^s^2^U_34_ tRNA modification. (b) Growth phenotype of 40-day-old wild-type (Col-0), *acl5, acl5 rol5, acl5 trm9*, and *acl5 alkbh8* mutant plants. Bar = 5 cm. (c) Plant height comparison between wild-type (Col-0) and mutant plants in the Col-0 background. Plants were grown in rockwool for 40 days. Mean ± SE, *n* = 5.

In the mcm^5^s^2^U tRNA modification pathway (Fig. 3a), the cytosolic thiouridylase ROL5 forms a functional complex with CTU2, while TRM9 functions as a methyltransferase in mcm^5^U formation. We thus tested whether the mutants of these genes have similar effects on *acl5* and found that both T-DNA insertion mutants of *ROL5* (Leiber et al. 2010) and *TRM9* (Leihne et al. 2011) suppressed the *acl5* dwarf phenotype in a similar degree to *ctu2-2* (Fig. 3b and c). In contrast, a mutant of *ALKBH8*, which is involved in tRNA modification but not in the mcm^5^s^2^U biosynthesis pathway (Leihne et al. 2011), did not suppress *acl5* (Fig. 3b and c).

### Translation of uORF-GUS mRNAs of both *SACL3* and *LHW* is reduced by *ctu2*

**Figure 4:**
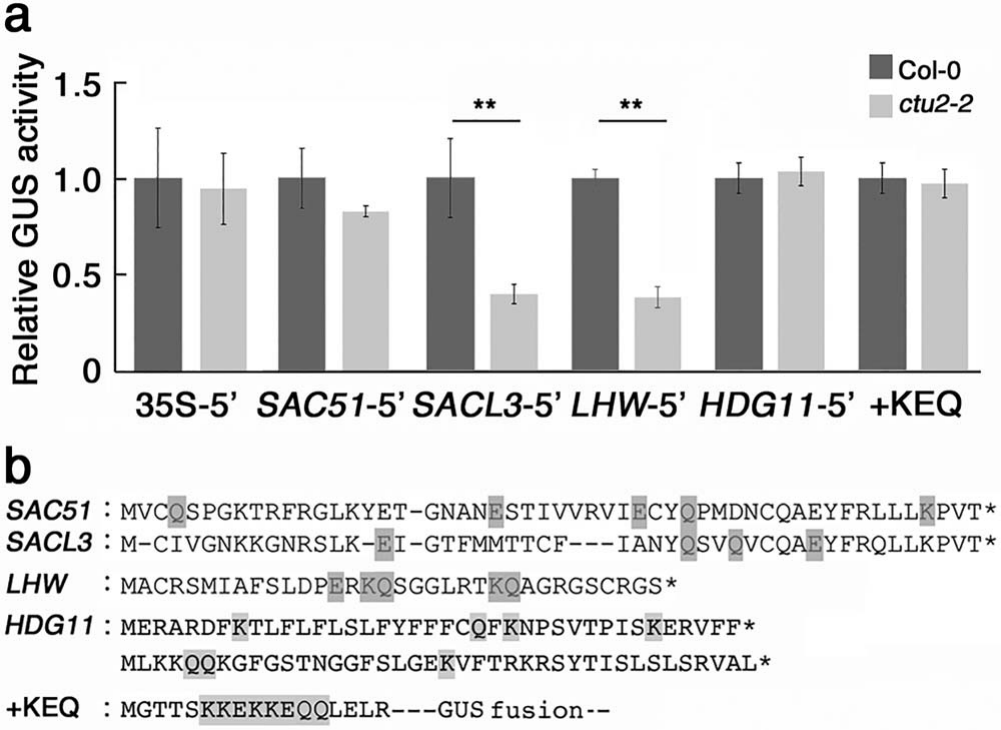
Effect of *ctu2* on the translation from GUS fusion transcripts. (a) Comparison of the GUS activity between Col-0 and *ctu2* 7-day-old seedlings carrying each GUS fusion construct. The 5′ leader regions of *SAC51, SACL3, LHW*, and *HDG11* transcripts were inserted between the CaMV 35S promoter and the GUS coding sequence of pBI121, shown as 35S-5′, respectively. +KEQ indicates the GUS translational fusion containing seven consecutive amino acids involving thiolated tRNAs. Data represent mean ± SE, *n* = 5. Asterisks indicate significant difference from Col-0 (^**^P < 0.01, Student’s *t*-test). (b) Amino acid sequences of uORFs of *SAC51, SACL3, LHW*, and *HDG11*, and an N-terminal partial sequence of the GUS translational fusion (+KEQ). The conserved uORFs of *SAC51* and *SACL3* are aligned with gaps. Amino acids involving thiolated tRNAs for translation are shaded.

Previous studies have shown that the suppression of the *acl5* phenotype by *suppressor-of-acl5* (*sac*) mutations so far identified is primarily attributable to uncoupling of mRNA translation of the *SAC51* family from thermospermine-dependent regulation by the defect in conserved uORFs or abnormalities in ribosomal components or assembly processes (Imai et al. 2008, Kakehi et al. 2015, Matsuo et al. 2022). We thus examined whether the *ctu2* mutation also enhances the translation of the *SAC51* family without thermospermine by using 5′ leader-GUS fusion reporters containing uORFs. When the GUS constructs were introduced into *ctu2* plants by crosses with each transgenic line, no significant effect of *ctu2* was observed on the GUS activity from the *SAC51* 5′ leader-GUS construct (Fig. 4a). Moreover, contrary to expectations, the GUS activity from the *SACL3* 5′ leader-GUS was significantly reduced in *ctu2* (Fig. 4a). On the other hand, *LHW* loss-of-function mutations also show effects as an *acl5* suppressor mutation (Katayama et al. 2015), and another conserved uORF is present within the *LHW* gene family (Hayden and Jorgensen 2007). Investigating the effect of the *ctu2* mutation on the *LHW* 5′ leader-GUS, we found a significant reduction in the GUS activity in *ctu2* (Fig. 4a). To examine the effect of *ctu2* on the translation of non-conserved uORF-containing mRNAs, we tested the GUS activity from the *HDG11* 5′ leader-GUS fusion construct. The 5′ leader of *HDG11*, encoding a homeodomain protein (Nakamura et al. 2006), is about 1.5 kb long and contains multiple non-conserved uORFs (Fig. 4b). We detected no effect of the *ctu2* mutation on the GUS activity from the *HDG11* 5′ leader-GUS construct (Fig. 4a). These uORFs contain four or more codons that involve thiolated tRNAs, namely AAA, CAA, and GAA (Fig. 4b). However, it remains unclear whether the number and arrangement of these codons correlate with the effects on GUS reporter translation in the mutant. We further examined the impact of the *ctu2* mutation on the GUS activity from a GUS translational fusion containing seven consecutive AAA/CAA/GAA codons inserted immediately downstream of the start codon but found no significant effect on the GUS activity in *ctu2* (Fig. 4a).

**Figure 5:**
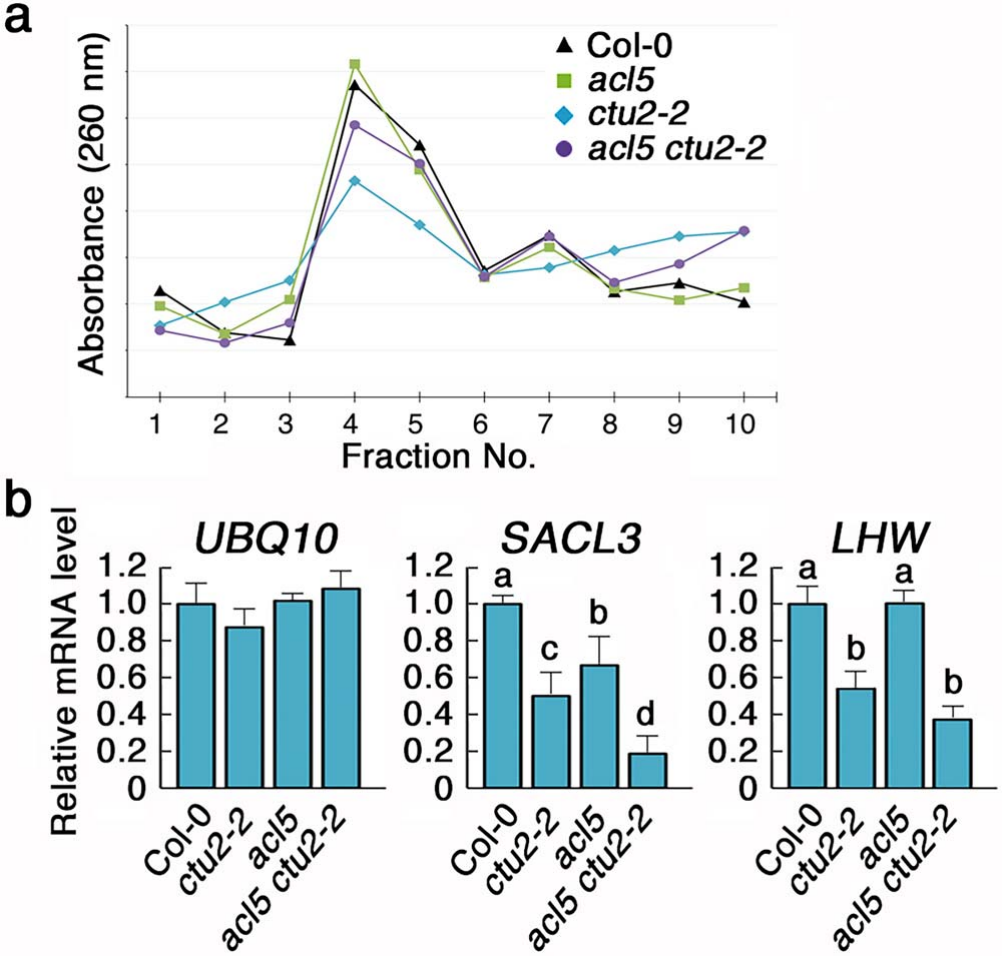
Polysome fractionation and qRT-PCR analysis. (a) Polysome profiles of wild-type (Col-0), *acl5, ctu2-2*, and *acl5 ctu2-2* seedlings. (b) Transcript levels of *UBQ10, SACL3*, and *LHW* in polysome fractions No. 9 + 10 shown in (a). The levels were normalized to the *ACT8* transcript level and set to 1 in Col-0. Means ± SE, *n* = 6. Different letters indicate significant differences at *P* < 0.05 by ANOVA.

Based on the above results, we hypothesized that the suppression of the *acl5* phenotype by the *ctu2* mutation is more likely due to the decreased translation of *LHW* rather than the effects on the translation of *SAC51* family genes. To confirm further the translation activity of *LHW* in *ctu2*, we prepared the polysome fractions by sucrose density gradient centrifugation (Fig. 5a) and quantified the *LHW* mRNA level. The results showed a clear reduction in the *LHW* mRNA in the polysome fraction of *ctu2* (Fig. 5b). The amount of the *SACL3* mRNA was also reduced in the polysome fraction of the mutant (Fig. 5b).

**Figure 6:**
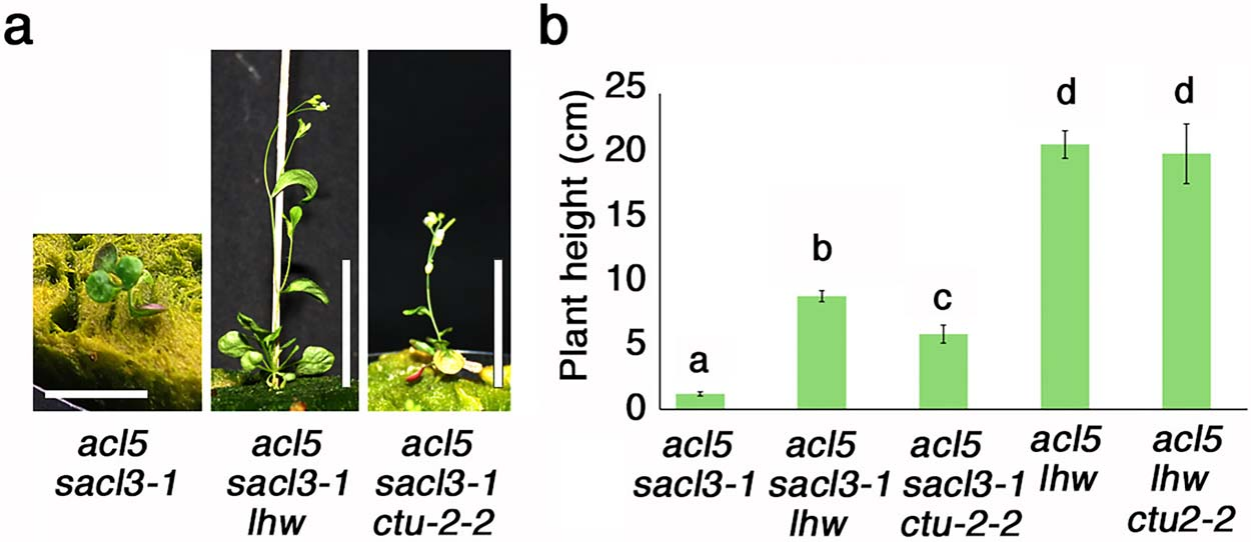
The tiny plant phenotype of *acl5 sacl3-1* is partially suppressed by *ctu2* and *lhw*. (a) Growth phenotype of 35-day-old *acl5 sacl3-1, acl5 sacl3-1 lhw*, and *acl5 sacl3-1* plants. Bars = 1 cm. (b) Plant height comparison between each mutant line. Plants were grown in rockwool for 35 days. Mean ± SE, *n* = 5. Different letters indicate significant differences at *P* < 0.05 by ANOVA.

The dwarf phenotype of *acl5* is exacerbated by the loss-of-function mutation in *SACL3*, resulting in very small plants (Cai et al. 2016), while *lhw* mutations partially recover this phenotype (Fig. 6a) (Katayama et al. 2015). These phenotypes of higher-order mutants involving *acl5* and knockouts of *SAC51* and *LHW* families reflect that xylem proliferation promoted by the *LHW* family is antagonized by the *SAC51* family in a thermospermine-dependent manner. We examined whether *ctu2* has the same suppressive effect as *lhw* on *acl5 sacl3* and observed the growth recovery in *acl5 sacl3 ctu2*, although not to the same extent as in *acl5 sacl3 lhw* (Fig. 6a and b). On the other hand, the growth recovery of *acl5* plants by *lhw* was not enhanced in *acl5 lhw ctu2* triple mutants (Fig. 6b).

### *ctu2* shows increased sensitivity to thermospermine

**Figure 7:**
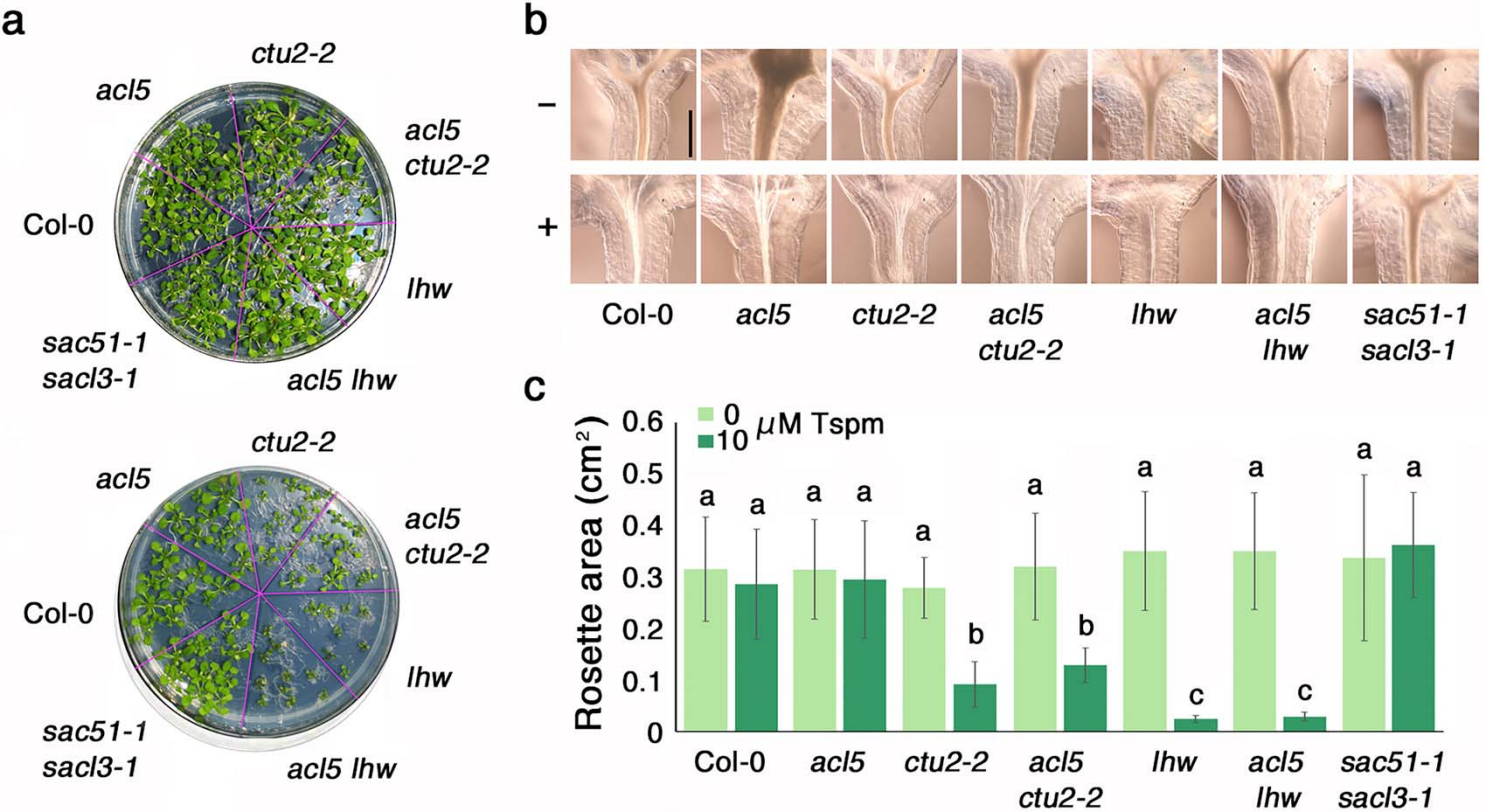
*ctu2* is hypersensitive to thermospermine. (a) Growth phenotype of 14-day-old wild-type (Col-0) and mutant seedlings grown without (upper panel) or with (lower panel) 10 *μ*M thermospermine. (b) Vascular phenotype in hypocotyls of 14-day-old wild-type (Col-0) and mutant seedlings grown without (−) or with (+) *10 μM* thermospermine. Samples were cleared with chloral hydrate. Scale bar, 0.5 mm. (c) Rosette areas of 14-day-old seedlings grown without or with 10 *μ*M thermospermine*.* Mean ± SE, *n* = 10. Different letters indicate significant differences at *P* < 0.05 by ANOVA.

While double loss-of-function mutants of *SAC51* and *SACL3* coding sequences are highly insensitive to thermospermine (Cai et al. 2016), *lhw* mutants show significant sensitivity to external thermospermine, with seedling growth being severely inhibited (Xu et al. 2025). We investigated whether the *ctu2* mutation also exhibits similar increased sensitivity. Both the *ctu2* single mutant and the double mutant with *acl5* showed higher sensitivity to thermospermine than wild-type seedlings, although not as pronounced as in *lhw*. In these mutants, leaf growth was severely inhibited (Fig. 7).

## Discussion

Our previous analyses of *acl5* suppressor mutants and their responsible genes have revealed a role of specific ribosomal proteins in thermospermine-dependent mRNA translation of the *SAC51* family. These include, RPL10, RPL4, and RACK1 (Imai et al. 2008, Kakehi et al. 2015). Based on these studies, it had been proposed that such suppression might result from a reduced requirement for thermospermine in uORF-mediated translational regulation of the *SAC51* family. In this study, we demonstrated that loss of thiolation of uridine at the wobble position of tRNA, corresponding to the first nucleotide of the anticodon, can partially suppress the *acl5* phenotype. Contrary to our expectations, GUS reporter assays revealed no significant enhancement of *SAC51* translation in the *ctu2* mutant background, while translation of *SACL3* was instead reduced. Notably, members of the *LHW* family also harbor conserved uORFs that are distinct from those found in the *SAC51* family. Using a 5′ leader–GUS fusion, we found that the *ctu2* mutation negatively affected translation of *LHW*. In the auxin-induced vascular xylem differentiation, LHW functions as a key transcription factor and positively regulates thermospermine biosynthesis through activation of *ACL5* expression. In turn, thermospermine promotes translation of the *SAC51* family, which interferes with the function of LHW and consequently feeds back negatively on xylem differentiation (Katayama et al. 2015, Vera-Sirera et al. 2015). Therefore, the partial suppression of excessive xylem differentiation in *acl5* by *ctu2* deficiency is most plausibly explained by reduced translation of *LHW* itself. A recent study has revealed that thermospermine interacts with a specific methylated base m^3^U2952 in the 25S rRNA, which counteracts the inhibitory effect of the uORFs on the translation of the *SAC51* family, while enhancing the inhibitory effect of the uORFs on the translation of the *LHW* family (Ko et al. 2026). It is likely that both translation processes are negatively affected in *ctu2*.

A loss-of-function mutation in *CTU2* has previously been reported in *A. thaliana*, where it causes reduced lateral root formation but does not result in severe developmental defects (Philipp et al. 2014). We observed that *ctu2* also causes a reduction in the looped pattern of cotyledon veins, like that observed in *lhw* (Ohashi-Ito and Bergmann 2007). In other organisms, mutations in *CTU2* or tRNA thiolation have been associated with accumulation of misfolded proteins, growth retardation, and increased stress sensitivity (Dewez et al. 2008, Fernández-Vázquez et al. 2013, Shaheen et al. 2019, Kojic et al. 2021, Zhang et al. 2025), yet no characteristic features have been identified regarding the frequency or distribution of the three codons decoded by thiolated tRNAs in target mRNAs. In yeast, reporter assays containing tandem target codons showed a modest reduction in translation in tRNA thiolation mutants (Damon et al. 2015), whereas in our analogous experiments such effects were not detectable. Our findings instead suggest that tRNA modification may play an important role in translational regulation mediated by highly conserved uORFs. Defects in tRNA modification are known to induce ribosome pausing at the A site, and accumulating evidence indicates that tRNA modifications primarily regulate translational speed rather than accuracy (Rezgui et al. 2013, Zinshteyn and Gilbert 2013, Nedialkova and Leidel 2015). Reduced translational speed at specific peptide sequences can have profound consequences for protein folding. In the uORF-containing genes examined here, particularly *SACL3* and *LHW*, where downstream main ORF translation was reduced, uORF-mediated regulation may be disrupted by mutation-induced alterations in translational elongation dynamics.

Furthermore, because uORFs are much shorter than main coding sequences, mutations affecting decoding of specific codons may have a disproportionately strong impact on uORF-mediated translational control. We observed different degrees of translational effects downstream of the 5′ leaders of *SAC51* and *SACL3*, both of which contain highly conserved uORFs (Fig. 4). This might be attributable to subtle differences in codon composition or spacing within their respective uORFs, which could influence elongation sensitivity. A broader and more detailed analysis will be required to fully understand genes regulated by uORFs in the context of tRNA modification defects.

In summary, this study demonstrates that, unlike previously identified ribosome-related suppressor mutations of *acl5*, tRNA modification mutations do not enhance translation of *SAC51* family mRNAs but instead reduce their translation and decrease *LHW* translation, thereby leading to phenotypic recovery. We have further identified thermospermine-insensitive mutants that display almost no repression of xylem formation by exogenous thermospermine, and their causative genes include those encoding enzymes responsible for rRNA methylation and pseudouridylation (Saraumi et al. 2025). These mutations also affect uORF-mediated translation of the *SAC51* family. Taken together, the molecular action of thermospermine may be multifaceted and involve different RNA modifications. Loss of mcm^5^s^2^U modification has been shown in fungi and other systems to cause ribosome pausing at specific codons in a context-dependent manner (Rezgui et al. 2013, Nedialkova and Leidel 2015, Schaffrath and Leidel 2017, Agris et al. 2018, Johansson et al. 2018). Our results extend these findings by demonstrating, for the first time in plants, that tRNA thiolation contributes to uORF-mediated translational regulation of key developmental regulators. It is conceivable that future studies will uncover many cases in which abnormal translation of uORF-regulated mRNAs represents a direct cause of phenotypes associated with defects in tRNA modification.

## Materials and Methods

### Plant material and growth conditions

*A. thaliana* wild-type accessions Columbia (Col-0) and Landsberg erecta (L*er*) were used. *acl5* (L*er*) was previously isolated (Hanzawa et al. 1997), and *acl5* (Col-0) was obtained after more than eight backcrosses of *acl5* (L*er*) to Col-0. The *sac502* (*ctu2-3*) *acl5* mutant was isolated from EMS-mutagenized *acl5* (L*er*) seeds by selecting individuals showing recovery of stem elongation. T-DNA insertion mutants, *ctu2-2* (GK-686B10*-*022973), *rol5-2* (GK-709D04*-*022875), *trm9* (SALK_135308), and *alkbh8-2* (SALK_094502), were obtained from the Arabidopsis Biological Resource Center. Primers used for PCR-based T-DNA detection and genotyping are listed in Supplementary Table S1.

Seeds were surface-sterilized with commercial bleach containing 5% sodium hypochlorite with 0.01% Triton X-100 for 3 min and rinsed three times with sterile water. Seeds were sown on agar plates (pH 5.8) containing 0.8% agar, 3% sucrose, and Murashige–Skoog (MS) nutrients (Wako, Tokyo, Japan), or on rockwool surrounded by vermiculite, and grown at 22°C under a 16-h-light/8-h-dark photoperiod.

### DNA extraction

Genomic DNA was extracted by grinding a single *Arabidopsis* leaf in 200 *μ*L of DNA extraction buffer [0.2 M Tris–HCl (pH 8.0), 2.5 mM EDTA, 0.25 M NaCl, 0.5% SDS]. The lysate was centrifuged at 15 000 rpm for 5 min, and 150 *μ*L of the supernatant was mixed with isopropanol and kept at −20°C for 10 min. After centrifugation at 15 000 rpm for 5 min, the pellet was washed with 70% ethanol and dried with vacuum. The pellet was dissolved in 50 *μ*L distilled water and centrifuged at 15 000 rpm for 1 min. Two *μ*L of the supernatant was used for PCR. PCR was performed using Ex Premier DNA Polymerase (Takara, Kyoto, Japan) following the manufacturer’s instructions.

### Chromosome mapping and identification of point mutation

*sac502 acl5* (L*er*) was crossed with *acl5* (Col-0), and F2 individuals showing restored stem elongation were selected for chromosome mapping using Col-0/L*er* polymorphic markers (Tanaka et al. 2020). Whole-genome sequencing was outsourced to Bioengineering Lab (Sagamihara, Japan). Genotyping of the *sac502* mutation after sequence determination was performed by PCR using primers listed in Supplementary Table S1.

### GUS fusion constructs and plant transformation

Transgenic lines carrying the CaMV 35S promoter-*SAC51* or *SACL3* 5′ leader-*GUS* fusion were described previously (Kakehi et al. 2015, Matsuo et al. 2022). The *LHW* 5′ leader-*GUS* construct was generated by inserting a fragment of the *LHW* 5′ leader region, which was amplified from genomic DNA by PCR and digested with *Xba*I and *Bam*HI, between the CaMV 35S promoter and the *GUS* coding sequence of pBI121 (Clontech, CA, USA). For constructing the *GUS* translational fusion with seven consecutive amino acids, which involves thiolated tRNAs in translation, restriction sites of *Spe*I and *Xho*I were introduced immediately downstream of the start codon of the *GUS* coding sequence by PCR-based replacement of the 35S promoter-*GUS* gene in pBI121. Primers used for T-DNA construction are listed in Supplementary Table S1.

T-DNA constructs were introduced into the wild-type Col-0 genome by the floral dip method *(*Clough and Bent 1998) using *Agrobacterium tumefaciens* strain GV3101. Transgenic lines with 3:1 segregation for kanamycin-resistant phenotype were chosen as single-copy T-DNA insertion lines and further crossed with *ctu2-3* to produce those in the mutant background.

### GUS reporter assay

Ten-day-old seedlings grown on kanamycin-containing MS medium (50 *μ*g/mL) were ground in 100 *μ*L TE buffer and centrifuged at 15 000 rpm. Five *μ*L of the supernatant was mixed with 45 *μ*L GUS reaction buffer [50 mM Na₂HPO₄–NaH₂PO₄ (pH 7.0), 10 mM EDTA, 0.1% SLS, 10 mM 2-mercaptoethanol, 0.1% Triton X-100, 1 mM 4-methylumbelliferyl-β-D-glucuronide hydrate] and incubated at 37°C for 1 h. The reaction was stopped by adding 450 *μ*L of 0.2 M Na₂CO₃. Fluorescence was measured with an RF-spectrofluorophotometer (Shimadzu, Kyoto, Japan) at 365 nm excitation and 455 nm emission. Protein concentrations were determined by mixing 20 *μ*L of the extract with 1 mL of Bradford reagent (Bio-Rad, CA, USA).

### RNA extraction and quantitative RT-PCR

Ten-day-old seedlings grown on MS medium were ground in liquid nitrogen, and total RNA was extracted and purified using the SDS–phenol method. First-strand cDNA synthesis was performed using the PrimeScript II 1st Strand cDNA Synthesis Kit (Takara) according to the manufacturer’s instructions. The resulting cDNA was diluted 100-fold and used as a template for real-time PCR with the KAPA SYBR Fast qPCR Kit (KAPA Biosystems, MA, USA) on a Thermal Cycler Dice Real Time System Lite (Takara). *ACT8* was used as the internal control. Primers used for PCR are listed in Supplementary Table S1.

### Light microscopy

For sectioning, samples of hypocotyls and stems were fixed overnight in FAA (50% ethanol, 5% formaldehyde, 5% acetic acid) and dehydrated through an ethanol series. Samples were embedded in Technovit 7100 (Heraeus Kulzer, Wehrheim, Germany), sectioned at 10 *μ*m, and stained with 0.1% toluidine blue. For tissue clearing, 14-day-old seedlings grown on MS medium were fixed overnight in ethanol:acetic acid (9:1, v/v) and then incubated in clearing solution (chloral hydrate:glycerol:water = 8:1:2) overnight. Samples were observed under a light microscope ECLIPS 80i (Nikon, Tokyo, Japan).

### Polysome preparation

Polysome fractions were prepared according to Lecampion et al. (2016) with some modifications. Fourteen-day-old seedlings grown on MS plates were ground in liquid nitrogen and homogenized in polysome buffer [100 mM Tris–HCl (pH 8.4), 50 mM KCl, 25 mM MgCl₂, 5.26 mM EGTA, 0.005% Triton X-100, 50 *μ*g/mL cycloheximide]. After centrifugation at 15 000 rpm for 20 min, the supernatant was loaded onto 20%–50% (w/v) sucrose gradients and centrifuged at 32 000 rpm for 3 h at 4°C using an SW41 rotor (Beckman, CA, USA). The gradient was fractionated into 10 equal fractions, and RNA concentrations were measured using a NanoDrop spectrophotometer (Thermo Fisher Scientific, MA, USA). The bottom polysome-enriched fractions were extracted with phenol–chloroform and precipitated with an equal volume of isopropanol in the presence of 50 *μ*g/mL glycogen. Pellets were washed with 70% ethanol, dissolved in 5 *μ*L distilled water, and the entire sample was subjected to RT-PCR.

### Thermospermine treatment and quantification of rosette areas

Seeds were sown on MS plates containing 10 *μ*M thermospermine, and rosette areas of 14-day-old seedlings were analyzed. Quantification of rosette area was performed using ImageJ with the Rosette Tracker plugin (Vylder et al. 2012).

## Supplementary Material

Supplementary_table_S1_pcag055

## Data Availability

All data presented in this study are available within the article and supplementary materials.
